# Neuropathic Pain: Mapping the miRNA Landscape

**DOI:** 10.3390/ncrna12020013

**Published:** 2026-04-06

**Authors:** Mario García-Domínguez

**Affiliations:** Facultad de Educación, Universidad Alfonso X El Sabio (UAX), Avenida de la Universidad, 1, Villanueva de la Cañada, 28691 Madrid, Spain; marigado@uax.es

**Keywords:** miRNA, biomarkers, prognostic evaluation, regulatory networks, neuropathic pain, clinical outcomes

## Abstract

Neuropathic pain represents a complex, prolonged pain state arising from lesions within the somatosensory nervous system. Despite significant advances in elucidating its pathophysiology, current therapeutic approaches remain largely symptomatic and frequently inadequate. MicroRNAs, a class of small non-coding RNAs that regulate gene expression post-transcriptionally, have recently emerged as critical modulators of neuronal excitability, neuroinflammation, and synaptic plasticity, which are crucial processes in the development and maintenance of neuropathic pain. This review summarizes the current evidence linking specific miRNAs to the onset and maintenance of neuropathic pain, with an emphasis on their roles in peripheral and central sensitization. The potential of miRNA-based biomarkers for diagnosis and prognostic evaluation is also highlighted. A thorough understanding of the complex miRNA regulatory networks underlying neuropathic pain could facilitate the development of novel, mechanism-based therapies and ultimately improve clinical outcomes.

## 1. Introduction

According to the International Association for the Study of Pain (IASP), pain is defined as an “unpleasant sensory and emotional experience associated with, or resembling that associated with, actual or potential tissue damage” [1,2]. Pain can be classified into distinct subtypes based on a range of characteristics, including its underlying origin [3]. Nociceptive pain arises from the activation of nociceptors due to actual or potential damage to non-neural tissues [4]. Nociplastic pain is characterized by altered nociceptive processing in the absence of clear evidence for tissue damage, often resulting from pathological lesions within the somatosensory system [5]. Neuropathic pain, in contrast, is attributable to a definitive disease or lesion affecting the somatosensory nervous system.

Neuropathic pain may result from direct injury to components of the nervous system [6]. Clinically, patients report numerous distinctive sensory phenomena, including burning sensations, cold or electric-like dysesthesias, tingling, paresthesia, numbness, and increased somatosensory sensitivity [7]. These manifestations may be accompanied by allodynia and hyperalgesia [8]. The pathophysiology of neuropathic pain involves complex alterations in the peripheral nervous system (PNS) and central nervous system (CNS) [9,10,11]. Peripheral sensitization encompasses hyperexcitability of damaged nerve fibers, increased spontaneous neuronal activity, and heightened responsiveness to several stimuli [9,10]. Central sensitization is characterized by amplification of nociceptive signal transmission and processing within the CNS structures, culminating in enhanced pain perception [9,11].

Neuropathic pain may arise as a secondary consequence of many underlying conditions, like diabetic peripheral neuropathy (DPN), chemotherapy-induced peripheral neuropathy (CIPN), spinal cord injury, postherpetic neuralgia, trigeminal neuralgia, multiple sclerosis, and HIV-associated neuropathy [6,7]. Reported prevalence rates of neuropathic pain have varied substantially across epidemiological studies, largely due to methodological heterogeneity in assessment approaches and population characteristics. Nevertheless, recent large-scale studies provide more robust estimates. For instance, analysis of the UK Biobank cohort estimated the prevalence of neuropathic pain at approximately 9.2% [12]. Additional population-based evidence studies suggest a prevalence range between 6.9% and 10% [13]. In Spain and other European countries, reported prevalence rates are generally within the 6–8% [14,15].

Despite decades of research into its underlying pathophysiological mechanisms, the clinical management of neuropathic pain remains a major therapeutic challenge. Current pharmacological treatments, including anticonvulsants (such as gabapentinoids) [16], antidepressants (like tricyclic antidepressants and serotonin-norepinephrine reuptake inhibitors) [17], and opioids [18] often provide limited analgesic benefits, with clinically meaningful pain reduction achieved in approximately 30–50% of patients [19]. In addition, these treatments are often associated with dose-limiting systemic adverse effects, development of tolerance, and, in the case of opioids, a significant risk of dependence and misuse [20]. These limitations highlight the pressing need for more effective, mechanism-based therapeutic strategies; thus, advancing our understanding of the molecular and cellular pathways underlying peripheral and central sensitization in neuropathic pain is critical for the rational design of targeted interventions that modulate maladaptive pain signaling rather than merely providing symptomatic relief.

Neuropathic pain arises from an intricate interplay of molecular and cellular mechanisms that amplify nociceptive signaling and sustain pathological excitability. Following nerve injury, sensory neurons undergo transcriptional and post-translational modifications in key ion channels, including voltage-gated sodium channels (VGSCs) and voltage-gated calcium channels (VGCCs), leading to lowered activation thresholds and enhanced ectopic firing [21,22]. Concurrently, transient receptor potential (TRP) channels, including TRPV1 and TRPA1, are upregulated at peripheral terminals, heightening thermal and mechanical sensitivity [23]. Activated microglia and astrocytes play an essential role in neuroimmune interactions by releasing several pro-inflammatory cytokines (IL-1β and TNF-α), chemokines (CCL2 and CX3CL1), and reactive oxygen and nitrogen species (ROS and RNS, respectively), which regulate channel phosphorylation, trafficking, and membrane localization, ultimately enhancing nociceptor hyperexcitability [24,25]. In the spinal dorsal horn, these mediators enhance glutamatergic transmission via NMDA receptor phosphorylation, suppress inhibitory GABAergic and glycinergic signaling, and increase local expression of neurotrophic factors, such as NGF and BDNF, which potentiate synaptic plasticity in second-order neurons [26,27]. This creates a self-reinforcing loop where glial-derived inflammatory signals perpetuate both peripheral sensitization and central hyperexcitability. Moreover, sustained neuroinflammation induces epigenetic remodeling in neurons and glia, altering ion channel transcription and receptor density, thereby stabilizing the chronic neuropathic pain state [28].

Over the past decade, non-coding RNAs (ncRNAs), mostly microRNAs (miRNAs), have emerged as pivotal regulators of gene expression and neuronal function, providing novel insights into the molecular mechanisms underlying neuropathic pain [29]. miRNAs are small (~22 nucleotides), conserved non-coding RNAs that regulate gene expression post-transcriptionally by binding to complementary sequences in target messenger RNAs (mRNAs), leading to mRNA degradation [30]. Each miRNA can regulate many mRNAs, and conversely, individual mRNAs can be targeted by multiple miRNAs, forming a complex regulatory network capable of finely tuning cellular processes [31]. Within the nervous system, miRNAs orchestrate key mechanisms such as neuronal excitability, synaptic plasticity, axonal regeneration, and neuroinflammation, processes intimately implicated in the initiation, maintenance, and chronification of neuropathic pain [32,33].

Experimental and clinical studies have demonstrated that dysregulation of specific miRNAs contributes to maladaptive changes in both peripheral and central neural circuits following nerve injury. In the PNS, altered miRNA expression can modulate ion channel function, inflammatory mediator release, and neuronal hyperexcitability, thus promoting peripheral sensitization [34]. Within the CNS, miRNAs modulate synaptic transmission, regulate microglial and astrocyte activation, and facilitate the potentiation of nociceptive pathways, all of which are key contributors to central sensitization [35,36].

Beyond their mechanistic roles, miRNAs have emerged as promising biomarkers and therapeutic targets for neuropathic pain [37]. Their stability in many biological fluids and disease-specific expression patterns facilitates non-invasive diagnostics, prognostics, and monitoring of therapeutic responses [38]. miRNA-based therapeutics (including mimics, inhibitors-antagomiRs-, and gene delivery systems) offer precise modulation of maladaptive molecular pathways, representing a shift toward mechanism-based precision medicine [39].

This review presents a comprehensive synthesis of current evidence implicating miRNAs in the initiation, maintenance, and modulation of neuropathic pain, with a particular focus on their roles in peripheral and central sensitization, neuroinflammation, and synaptic plasticity. It also examines the translational potential of miRNAs as biomarkers and therapeutic targets, highlighting recent advances in miRNA-based strategies and the challenges of clinical implementation. By integrating insights into the complex regulatory networks governed by miRNAs in neuropathic pain, the review aims to inform future research directions and facilitate the development of mechanism-based, precision therapies to mitigate chronic pain and improve patient outcomes.

Importantly, beyond simply cataloguing individual miRNAs linked to neuropathic pain, this review employs an integrative mechanistic framework that systematically connects miRNA dysregulation with molecular and cellular substrates of peripheral and central sensitization. In addition, this review appraises the translational robustness of existing evidence and delineates key methodological and clinical barriers that must be overcome to allow the implementation of miRNA-based precision therapeutics in clinical settings.

## 2. MicroRNAs: Characteristics and Functions

### 2.1. Biogenesis of miRNAs

**Figure 1:**
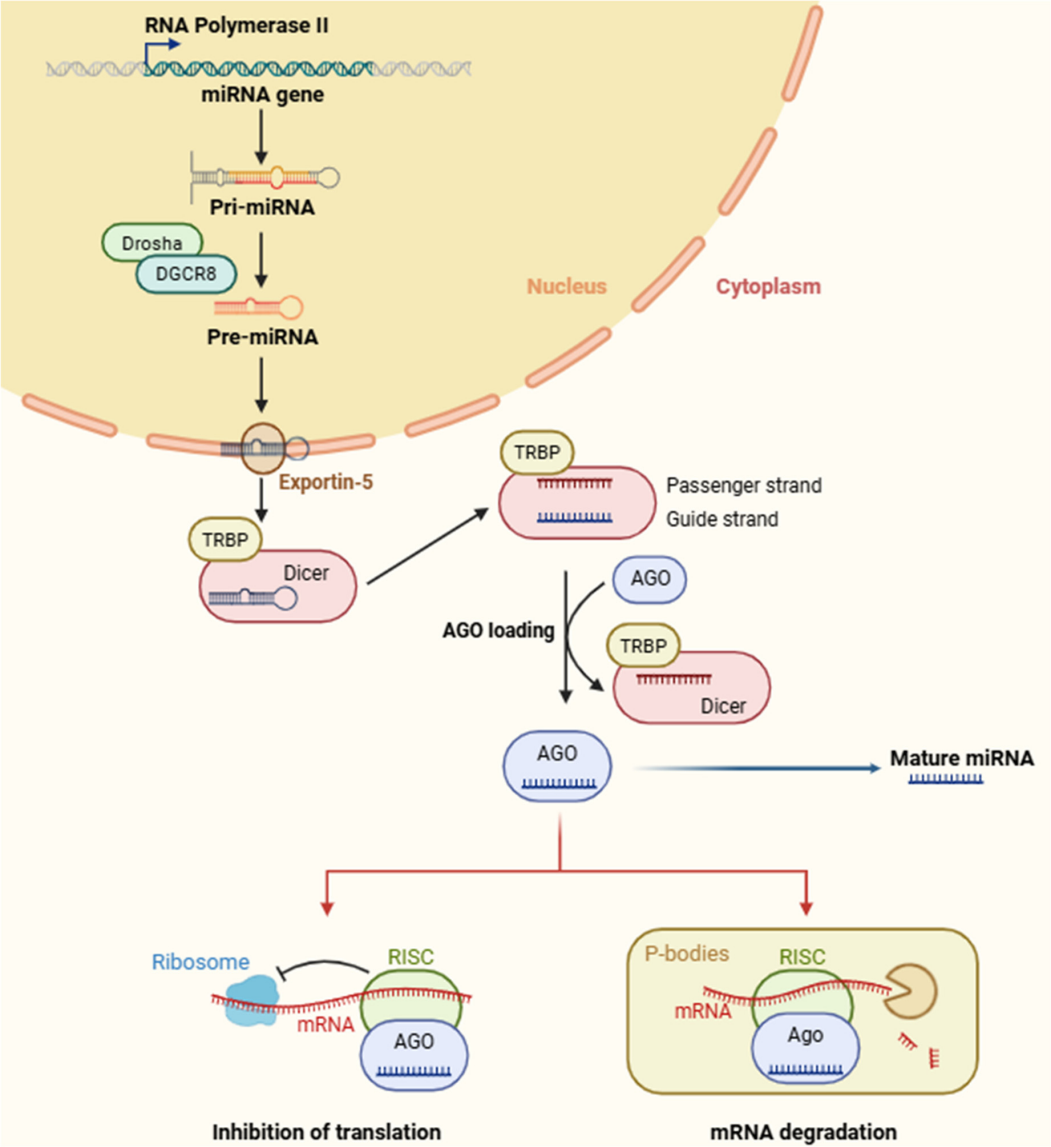
Biogenesis and effector functions of microRNAs. miRNA genes are transcribed by RNA polymerase II into pri-miRNAs, which are cleaved in the nucleus by the Drosha-DGCR8 complex to form pre-miRNAs. Pre-miRNAs are exported to the cytoplasm via Exportin-5/Ran-GTP and processed by Dicer/TRBP into miRNA duplexes. The guide strand is loaded onto AGO to assemble the RISC complex, which mediates translational repression or mRNA decay in P-bodies, depending on the miRNA-mRNA complementarity. Abbreviations: miRNA (microRNA), pri-miRNA (primary microRNA), pre-miRNA (precursor microRNA), DGCR8 (DiGeorge syndrome critical region 8), TRBP (TAR RNA-binding protein), Dicer (RNase III endonuclease), AGO (argonaute), RISC (RNA-induced silencing complex), and mRNA (messenger RNA).

The biogenesis of miRNAs is a regulated, multistep process that integrates nuclear and cytoplasmic events, allowing precise post-transcriptional control of gene expression (Figure 1). MiRNAs are encoded by independent genes, introns of protein-coding genes, or polycistronic clusters, and their transcription is predominantly carried out by RNA polymerase II, which generates capped and polyadenylated primary miRNA transcripts (pri-miRNAs) [40]. A subset of miRNAs, particularly those located within repetitive sequences or specific small RNA loci, may be transcribed by RNA polymerase III [41]. Pri-miRNAs are usually several hundred to thousands of nucleotides in length and contain characteristic stem-loop structures, which are fundamental for recognition by the processing machinery [42]. These transcripts may also contain regulatory elements including enhancer-associated regions, splice sites, or RNA-binding protein motifs that influence their stability, folding, and eventual processing efficiency [43].

Once synthesized, pri-miRNAs undergo initial nuclear processing by the microprocessor complex, composed of Drosha, an RNase III endonuclease, and DGCR8 (DiGeorge syndrome critical region 8), a double-stranded RNA-binding protein [44]. DGCR8 interacts with the pri-miRNA stem-loop, directing Drosha to the boundary between the single-stranded flanking regions and the double-stranded stem. Drosha then performs site-specific cleavage at the hairpin base, creating a 70-nucleotide pre-miRNA with a 2-nucleotide 3′ overhang [45,46], essential for recognition by nuclear export machinery. Auxiliary proteins (e.g., hnRNPs, Smads, p68/p72 helicases, and other RNA-binding factors) can modulate Drosha cleavage efficiency in response to cellular signals, stress, or developmental cues [47,48,49,50].

Pre-miRNAs are subsequently transported to the cytoplasm by exportin-5 (XPO5), a karyopherin-family nuclear transport receptor, which specifically detects the 3′ overhang and forms a ternary complex with Ran-GTP to provide energy and control, though some studies suggest Ran-GTP-independent export [51,52,53]. Proper regulation of this process is crucial, as disruptions in XPO5 expression or Ran-GTP gradients can impair miRNA maturation and gene regulatory networks [54].

In the cytoplasm, Dicer, an RNase III endonuclease, processes pre-miRNAs near the terminal loop to produce 21–25 nucleotide RNA duplexes containing the mature miRNA and the passenger (miRNA*) strand [55,56]. Dicer function is facilitated by cofactors such as TRBP or PACT, which enhance substrate recognition, stabilize Dicer-pre-miRNA interactions, and influence strand selection [57,58]. Strand selection is a critical regulatory step: the guide strand is incorporated into Argonaute (AGO) within the RNA-induced silencing complex (RISC), while the passenger strand is frequently degraded [59,60]. RISC assembly involves conformational rearrangements and ATP-dependent chaperone activity to ensure proper loading and orientation of the guide strand [61].

The miRNA-RISC complex locates target mRNAs through base-pairing, mainly between nucleotides 2–8 of the miRNA and complementary sequences in the 3′ UTR of target mRNAs [62]. Perfect or near-perfect complementarity typically triggers AGO2-mediated cleavage and degradation, more common in plants but also seen in specific animal targets [63]. Partial complementarity generally induces translational repression, deadenylation, and mRNA destabilization through cofactors including GW182, CCR4-NOT, and decapping enzymes [64,65].

Crucially, the biogenesis and function of miRNAs are context-dependent and highly dynamic, regulated by developmental stage, cellular state, and environmental signals [66]. Post-transcriptional modifications, including RNA editing (e.g., adenosine-to-inosine), 3′ uridylation or adenylation, and methylation, modulate miRNA stability, strand selection, and target specificity [67].

### 2.2. Mechanisms of Action of miRNAs

Through the mechanisms described in the following section, miRNAs play key roles in a broad spectrum of fundamental biological processes. miRNAs modulate cell proliferation and differentiation by controlling the expression of genes involved in cell cycle progression, lineage specification, and cellular maturation [68]. By coordinating repression or fine-tuning of multiple target mRNAs, miRNAs exert precise temporal and spatial control of gene regulation throughout embryogenesis and adult tissue homeostasis [69]. This regulation is essential for tissue homeostasis, enabling proper balance between self-renewal and differentiation, appropriate responses to developmental cues, and prevention of aberrant proliferative or differentiation programs [70].

In apoptosis, miRNAs act as either pro- or anti-apoptotic regulators by modulating genes involved in cell survival, mitochondrial integrity, and caspase activation [71]. They also regulate cellular stress responses to oxidative stress, hypoxia, endoplasmic reticulum stress, and DNA damage, promoting adaptation and survival under adverse conditions [72]. In immune regulation, miRNAs coordinate immune cell development, cytokine production, and resolution of inflammation, thereby maintaining immune homeostasis [73].

Beyond intracellular functions, miRNAs mediate intercellular communication. They are actively released into biological fluids, such as blood, saliva, urine, cerebrospinal fluid, and breast milk [74,75]. Extracellular miRNAs are highly stable, usually encapsulated in extracellular vesicles like exosomes and microvesicles [76,77,78], or linked to RNA-binding proteins (notably AGO) or lipoprotein complexes [79]. Once internalized by target cells, extracellular miRNAs modulate gene expression and influence cellular behavior, extending their regulatory role beyond the originating cell [80]. This communication is very important in immunosurveillance, tissue remodeling, tumor-stroma interactions, and metastatic niche formation [81,82,83], highlighting miRNAs as systemic signaling molecules [84].

Dysregulation of miRNAs is implicated in various diseases. In cancer, miRNAs may function as oncogenes or tumor suppressors, affecting cell cycle, apoptosis, angiogenesis, and metastasis [85]. Aberrant miRNA expression contributes to neurodegenerative disorders by influencing neuronal survival, synaptic plasticity, and neuroinflammation [86]. In cardiovascular disease, miRNAs regulate cardiac development, vascular integrity, and responses to ischemia [87]. Furthermore, disrupted miRNA-mediated immunomodulation can drive chronic inflammation and autoimmunity [88].

### 2.3. miRNA-Mediated Gene Regulation in the PNS and CNS: Implications for Neuronal Function

miRNAs in the CNS (Table 1) act as master regulators of neuronal architecture, synaptic plasticity, and intracellular signaling by targeting specific transcripts involved in cytoskeletal remodeling, receptor trafficking, and mitochondrial function [89]. miR-132 regulates dendritic arborization and spine morphogenesis by suppressing the GTPase-activating protein p250GAP, a negative regulator of Rac1 signaling. By reducing p250GAP levels, miR-132 promotes Rac1-mediated actin polymerization, resulting in augmented dendritic branching and spine density [90]. Additionally, miR-132 targets MeCP2, linking activity-dependent transcriptional programs to structural plasticity, thus coupling CREB-mediated transcriptional activation with cytoskeletal reorganization [91]. Through this dual targeting, neurons can integrate extracellular cues such as BDNF into both transcriptional and post-transcriptional programs that shape dendritic morphology and synaptic function.

miR-134, highly enriched in dendritic spines, exerts local translational repression of Limk1, a kinase that phosphorylates cofilin to regulate actin filament dynamics [92]. Fine-tuning Limk1 translation at individual spines allows miR-134 to control spine size and morphology, directly impacting synaptic strength and long-term potentiation (LTP) [93]. Activity-dependent NMDA receptor signaling can relieve miR-134-mediated repression, enabling rapid remodeling of dendritic spines in response to neuronal activity [94]. This mechanism exemplifies how miRNAs spatially restrict protein synthesis to coordinate cytoskeletal rearrangements critical for learning and memory [95]. miR-124 establishes and maintains neuronal identity in the CNS by repressing transcriptional repressors, including REST (RE1-Silencing Transcription factor) and Sox9 [96,97]. Inhibition of REST allows the expression of numerous neuron-specific genes involved in neurotransmission, cytoskeletal organization, and synaptic vesicle trafficking [98]. Furthermore, miR-124 targets RhoA, a regulator of actin dynamics, contributing to dendritic growth and elongation [99]. Through the integration of transcriptional repression and cytoskeletal modulation, this miRNA ensures proper neuronal differentiation and the maintenance of mature neuronal morphology [100].

miR-138 governs dendritic spine morphology and synaptic function by suppressing APT1 (acyl-protein thioesterase 1), which modulates the palmitoylation of several cytoskeletal and synaptic proteins, and RhoC, a member of the Rho GTPase family controlling actin dynamics. By repressing RhoC, miR-138 reduces actomyosin contractility, allowing spine expansion and stabilization [101]. miR-181 targets transcripts involved in dendritic protein translation and synaptic modulation. By repressing SIRT1, a deacetylase affecting transcription factors and cytoskeletal proteins, miR-181 promotes dendritic branching and proper localization of synaptic proteins [102]. miR-9 is crucial for dendritic development and spine formation in CNS neurons. This miRNA inhibits REST and transcription factors such as TLX, facilitating neuronal differentiation and dendritic complexity. By regulating Rho GTPase signaling and cytoskeletal effectors, miR-9 ensures accurate dendritic arbor formation and contributes to activity-dependent plasticity [103].

miR-128 modulates CNS neuronal excitability and dendritic arborization by repressing epigenetic regulators (e.g., Phf6 and Ezh2), which influence cytoskeletal gene expression and synaptic receptor composition [104]. miR-125b regulates dendritic spine density and synaptic stability by controlling the translation of PSD-95 and other postsynaptic density proteins [105]. miR-29a/b contributes to dendritic maintenance and neuronal survival by repressing pro-apoptotic factors including Bcl2l2 and integrins involved in cytoskeletal remodeling, thus preserving dendritic integrity and synaptic stability under stress conditions [106,107]. miR-153 contributes to synaptic homeostasis via repression of α-synuclein (SNCA) and vesicular transport proteins, thereby preventing the accumulation of toxic proteins at synapses and maintaining efficient neurotransmitter release, which supports dendritic spine stability and synaptic plasticity [108,109]. Finally, miR-210 regulates dendritic mitochondrial function by targeting ISCU and additional mitochondrial genes, thus optimizing ATP production within dendritic compartments and modulating spine morphogenesis and activity-dependent structural plasticity [110].

In the PNS (Table 1), miRNAs exert precise control over axonal growth, guidance, regenerative capacity, and glial support via molecular mechanisms that are distinct from those operating in CNS neurons. miR-21 represses PTEN and SPRY2, negative regulators of the PI3K-AKT and MAPK signaling pathways. Targeting these inhibitory biomolecules enhances AKT-dependent cytoskeletal remodeling, promotes growth cone dynamics, and facilitates axonal regeneration [111]. In Schwann cells, miR-21 further modulates the expression of pro-apoptotic and growth-inhibitory genes, thus supporting myelination and remyelination during nerve repair [112]. miR-182 targets Rac1 and Cofilin in PNS neurons, critical regulators of actin filament assembly and disassembly in growth cones. Through modification of Rac1 and Cofilin activity, miR-182 ensures coordinated actin polymerization and microtubule stabilization, essential for accurate axonal guidance and elongation [113]. miR-431 regulates peripheral axonal regeneration by targeting Kremen1 and Sprouty2, inhibitors of Wnt and MAPK signaling, respectively. By blocking these target biomolecules, miR-431 promotes growth cone remodeling and potentiates regenerative signaling pathways. This includes the activation of β-catenin-mediated transcriptional programs that regulate cytoskeletal gene expression, thus facilitating axonal outgrowth and structural reorganization necessary for neuronal regeneration [114].

miR-9 is expressed in PNS neurons and targets MAP1B and Neurofilament L, cytoskeletal components involved in microtubule stabilization and axonal elongation. By fine-tuning MAP1B levels, miR-9 ensures proper axonal caliber and branching patterns, while suppressing excessive neurofilament accumulation that could impair growth cone motility [115]. miR-132 is expressed in developing DRG axons, with knockdown impairing and overexpression enhancing axon extension. It acts locally through the Ras GTPase activator Rasa1, connecting miR-132 to growth cone signaling and axon elongation [116].

miR-124 works in part by targeting Sox9 and PTBP1 in neurons and Schwann cells, promoting differentiation and blocking aberrant glial proliferation [117]. miR-195 promotes PNS regeneration by targeting BACE1, regulator of microtubule stability and local protein degradation, thereby promoting growth cone motility and axonal elongation [118].

Finally, miR-338, localized in CNS dendrites and PNS axons, targets mitochondrial transcripts including COX4I1, which encodes cytochrome c oxidase subunits essential for ATP production [119]. In CNS neurons, miR-338 modulates local dendritic energy supply to support spine morphogenesis and synaptic activity [120], whereas in PNS axons, miR-338 maintains adequate ATP levels for cytoskeletal remodeling and growth cone motility [121].

## 3. Role of miRNAs in Neuropathic Pain

In recent years, dysregulated miRNA expression has been identified in many experimental models of neuropathic pain. These microRNAs regulate pain signaling through the targeting of genes linked to neuroinflammation, ion channel expression, neurotransmitter release, glial activation, and neuronal excitability. Dysregulation of microRNA expression has been documented in peripheral nerves, dorsal root ganglia, the spinal cord, and supraspinal structures involved in pain processing following nerve injury. Understanding the specific miRNA-target interactions involved in neuropathic pain might provide novel insights into disease mechanisms and facilitate the development of more effective and personalized pain management strategies.

Table 2 provides a summary of all known miRNAs implicated in the development of neuropathic pain. For each miRNA, this table specifies the experimental species in which it was studied, the neuropathic pain model employed, the validated or predicted molecular targets, and the reported effects on pain-related behaviors or signaling pathways.

Across the wide range of experimental models summarized in Table 2, a clear mechanistic convergence emerges despite the apparent diversity of dysregulated miRNAs, indicating that miRNA-dependent regulation in neuropathic pain is not random but clustered around a limited number of core biological pathways.

The most prominent and consistently represented mechanism is neuroinflammation. Numerous miRNAs modulate the production of pro-inflammatory cytokines such as IL-1β, IL-6, and TNF-α or regulate glial activation, including miR-15 [124], miR-93 [143], miR-98 [145], miR-124 [153,154], miR-129 [160], miR-140 [173], miR-144 [178], miR-182 [190], miR-183 [192], miR-185 [194], miR-195 [199], miR-200a [202], miR-204 [205], miR-216 [210], miR-221 [212], miR-223 [214], miR-340 [222], miR-381 [225], miR-411 [230], miR-506 [240], miR-544 [244], and miR-1906 [251].

On the other hand, several miRNAs converge on innate immune receptors and adaptor proteins within Toll-like receptor (TLR) signaling cascades. TLR4 and TLR5 are regulated by miR-100 [147], miR-150 [183], miR-154 [185], and miR-1906 [251], while IRAK1 and TRAF6 are targeted by miR-146a [180,181] and miR-125 [155], and MyD88 by miR-185 [194]. Additional convergence is observed at the level of inflammasome activation and danger signals: miR-34c [141], miR-185 [194], and miR-223 [214] regulate NLRP3, whereas miR-129 [160], miR-141 [174], miR-142 [175], miR-193 [197], and miR-381 [225] suppress HMGB1. These regulatory events consistently feed into NF-κB signaling, which emerges as a recurrent downstream hub modulated either directly or indirectly by miR-100 [147], miR-145 [179], miR-200a [202], miR-506 [240], miR-543 [243], miR-665 [247], and miR-1906 [251], highlighting NF-κB-driven transcription as a key integrative node in miRNA-mediated neuropathic pain mechanisms.

Alongside NF-κB activation, the JAK/STAT pathway represents another major signaling node. miR-93 [143], miR-98 [145], miR-218 [211], miR-544 [244], and miR-489 [235] regulate STAT3-dependent transcription, linking cytokine signaling to central sensitization and sustained inflammatory responses. In addition, numerous miRNAs converge on the PI3K/AKT/mTOR axis, including miR-15 [124], miR-20 [128], miR-145 [179], miR-182 [190], miR-183 [191], miR-212 [208], and miR-1906 [251], while MAPK signaling is regulated by miR-101 [148], miR-133a [164], and miR-223 [215]. Wnt/β-catenin (miR-30 [137], miR-92 [142], miR-216 [210]) and Notch signaling (miR-151 [184]) further extend this pattern, supporting the concept that miRNAs function as higher-order modulators integrating inflammatory and plasticity-related cascades.

Beyond inflammatory signaling, a substantial subset of miRNAs regulates neuronal excitability by targeting ion channels and synaptic components. VGSCs are frequently targeted, including Nav1.3/SCN3A by miR-96 [144], miR-212 [207], and miR-384 [228], and Nav1.1/SCN1A by miR-719 [250]. Ca^2+^ channel subunits are modulated by miR-103 [150], K^+^ channels (Kv2.1) by miR-323 [218], purinergic receptors (P2X4R by miR-106 [151] and miR-133b [165]) and P2X7R by miR-187 [195], and TRPV1 (by miR-142 [176] and miR-199 [201]).

Importantly, repeated targeting of identical molecular nodes by distinct miRNAs underscores the presence of redundant regulatory layers that stabilize pathological signaling networks. Examples such as AKT3 (miR-15 [124], miR-20 [128], and miR-145 [179]), ZEB1 (miR-28 [135], miR-128 [158], and miR-200b/429 [232]), STAT3 (miR-93 [143], miR-98 [145], and miR-544 [244]), HMGB1 (miR-129 [160], miR-141 [174], miR-142 [175], miR-193 [197], and miR-381 [225]), TRAF6 (miR-146a [180,181], and miR-125 [155]), and RAP1A (miR-202 [203], miR-203 [204], miR-331 [220], miR-340 [222], and miR-590 [246]). This recurrent targeting pattern implies that miRNAs do not act as isolated regulators but rather form an interconnected network that reinforces key pro-nociceptive pathways.

## 4. miRNAs as Biomarkers of Neuropathic Pain

The utility of miRNAs as biomarkers for neuropathic pain derives from a combination of their intrinsic molecular stability, regulatory specificity, and accessibility in various biological matrices [252]. Unlike most RNA species, miRNAs show remarkable resistance to enzymatic degradation, which is largely attributable to their protection within extracellular vesicles, particularly exosomes and microvesicles, as well as their association with RNA-binding proteins such as AGO2 and high-density lipoproteins (HDLs) [78,253]. This protective encapsulation not only prevents ribonuclease-mediated cleavage but also facilitates long-range intercellular communication, facilitating miRNAs released from affected neurons or glial cells to be detected in peripheral biofluids including plasma, serum, cerebrospinal fluid, and even saliva [254,255]. Consequently, miRNAs are accessible through minimally invasive sampling, enabling longitudinal monitoring of disease progression or therapeutic response, a significant advantage over conventional invasive approaches such as nerve biopsies or repeated cerebrospinal fluid collection [253].

The measurement of miRNAs is achieved via molecular approaches offering exceptional sensitivity. Quantitative real-time polymerase chain reaction (qRT-PCR) allows absolute quantification with sub-femtomolar sensitivity, while high-throughput microarray platforms and next-generation sequencing (NGS) allow profiling of the miRNA transcriptome [256,257]. Library refinements in construction methodologies, normalization strategies, and digital PCR approaches have further enhanced reproducibility, enabling comparative studies with high confidence [258,259]. Disease-specific miRNA signatures have been identified in several neuropathic pain etiologies (Table 3).

Multiple studies have shown that miRNAs in accessible biofluids serve as informative biomarkers for CNS disorders such as Alzheimer’s disease [260], epilepsy [261], and brain tumors [262]. Cerebrospinal fluid miRNA panels differentiate separate pathological from control states, with diagnostic performance often improved by incorporating genetic risk factors, while disease-specific signatures capture underlying pathophysiological processes [263]. Analogously, neuropathic pain exhibits dysregulated miRNAs in some biological fluids (plasma, serum, cerebrospinal fluid, and urine) [264,265,266,267,268,269,270,271,272].

**Table 3:** Differentially expressed miRNAs in blood and other fluids as potential biomarkers for diagnosing and monitoring neuropathic pain conditions. Abbreviations: miRNA (microRNA); DPN (diabetic peripheral neuropathy); CIPN (chemotherapy-induced peripheral neuropathy); PHN (post-herpetic neuralgia); CRPS (complex regional pain syndrome); and TN (trigeminal neuralgia).

Neuropathy Type	Fluid	miRNA	Expression Change	References
DPN	Plasma Serum Cerbrospinal fluid Urine	miR-21	Upregulated	[264]
	Blood	miR-146a	Upregulated	[265]
	Plasma	miR-199a-3p	Upregulated	[266]
	Serum	miR-499a	Genotype-specific (polymorphism-related)	[267]
CIPN	Plasma	miR-22-3p miR-23a-3p miR-24-3p	Upregulated	[268]
	Plasma	miR-3184-5p	Downregulated	[269]
PHN	Serum	mi-34c-5p miR-107 miR-127-5p miR-486-3p miR-892	Upregulated	[270]
CRPS	Blood	miR-31-5p miR-184 miR-296-3p miR-302-3p miR-1264	Downregulated	[271]
		miR-184	Upregulated	
TN	Serum	miR-34a miR-132-3p miR-146b-5p miR-155-5p miR-197 miR-202 miR-212 miR-384 miR-520b miR-523 miR-603 miR-661 miR-888	Upregulated	[272]

Definitely, systematic profiling of miRNAs in minimally invasive samples from neuropathic pain patients could identify disease-specific biomarker panels, enabling diagnosis, patient stratification, and treatment monitoring, and translating CNS miRNA insights into clinically actionable indicators of neuropathic pain pathophysiology.

On the other hand, integrating miRNA profiling with other high-dimensional omics modalities (such as transcriptomics, proteomics, and metabolomics) enables the development of multidimensional biomarker panels with upregulated sensitivity, specificity, and predictive performance compared to single-molecule measurements [273]. These signatures allow patient stratification based on disease severity, prediction of treatment responsiveness, and longitudinal monitoring of neuropathic pain progression [274].

Despite the promise of miRNAs as biomarkers, several technical and biological challenges remain. Patient-specific differences in miRNA expression, shaped by demographic factors, genetic background, and comorbidities, pose challenges for the accurate interpretation of circulating miRNA measurements [275]. Pre-analytical variables, including sample collection methods, storage conditions, RNA extraction protocols, and detection platforms, introduce further variability, requiring strict standardization [276]. Moreover, the pleiotropic properties of many miRNAs, which concurrently modulate multiple gene networks across diverse tissues, might reduce the specificity of individual markers for neuropathic pain, potentially limiting their applicability [277]. Advancements in standardized procedures, in silico deconvolution frameworks, cohort studies, and validation in separate populations are progressively alleviating these challenges.

## 5. Conclusions

miRNAs have emerged as pivotal regulators in neuropathic pain, modulating multiple genes involved in nociceptive signaling, neuroinflammation, and synaptic plasticity. Their capacity to simultaneously target diverse mRNAs allows them to orchestrate complex molecular networks that contribute to the initiation and maintenance of neuropathic pain. Dysregulated expression of some miRNAs in PNS and CNS has been shown to promote nociceptor sensitization, glial activation, and maladaptive neuronal plasticity.

Dysregulated miRNA profiles in neural tissues and biological fluids may function as potential biomarkers for diagnostic and prognostic indicators of neuropathic pain. Due to their biological stability, specificity, and accessibility in serum or cerebrospinal fluid, they make promising tools for non-invasive detection and monitoring of disease progression or therapeutic response. Furthermore, modulation of miRNA activity constitutes a novel therapeutic approach. Preclinical studies employing miRNA mimics or antagomirs have demonstrated significant attenuation of pain-related behaviors and neuroinflammatory responses, highlighting the translational potential of miRNA-based therapeutic strategies. For example, modulation of NRF2, a regulator implicated in the development and maintenance of neuropathic pain, has been linked to reduced nociceptive sensitization [187,237,278].

Despite these promising findings, several limitations should be considered. As with any narrative review, the interpretation of current evidence is limited by the heterogeneity in experimental models, methodologies, and analytical approaches across studies. Differences in species, injury paradigms, tissue sampling, and miRNA detection techniques may contribute to variability in reported miRNA profiles, which limits direct comparisons and the generalization of results. Furthermore, most of the current evidence derives from preclinical animal models, and therefore the translational relevance of specific miRNA signatures to human neuropathic pain conditions remains incompletely understood. Another important limitation lies in the complexity of miRNA-mediated regulation itself. A single miRNA can target multiple genes across different pathways, while individual genes may be regulated by multiple miRNAs. This intricate regulatory network makes it challenging to attribute specific functional outcomes to individual miRNAs and underscores the need for integrative approaches combining transcriptomics, proteomics, and functional validation.

A more comprehensive elucidation of specific miRNA-gene regulatory networks and their dynamic modulation in neuropathic pain will be essential for translating these findings into effective therapeutic strategies. For instance, a deeper understanding of how exercise influences these molecular pathways may provide valuable insight into its capacity to alleviate neuropathic pain symptoms [279]. Overall, miRNAs act as a crucial nexus linking gene regulation, neuroimmune signaling, and neuronal plasticity, providing unique opportunities to uncover pain mechanisms and to facilitate the development of innovative, targeted interventions.

Finally, future research should focus on the systematic characterization of miRNA expression profiles in well-defined clinical cohorts of patients with neuropathic pain, coupled with functional studies to elucidate their biological significance. Longitudinal studies evaluating circulating miRNAs as biomarkers for disease progression and treatment response would also provide valuable translational insights. Additionally, targeted experimental approaches (such as single-cell transcriptomics and CRISPR-based gene regulation) could help clarify the causal role of specific miRNAs within nociceptive circuits. Furthermore, controlled preclinical and clinical studies investigating how interventions such as physical exercise modulate miRNA-gene networks might characterize novel non-pharmacological strategies for neuropathic pain management.

## Figures and Tables

**Table 1:** miRNA-mediated regulation of cytoskeletal dynamics, synaptic plasticity, and axonal development in CNS and PNS. Abbreviations: CNS (central nervous system), PNS (peripheral nervous system), Rac1 (Ras-related C3 botulinum toxin substrate 1), MeCP2 (methyl-CpG-binding protein 2), LIMK1 (LIM domain kinase 1), REST (RE1-silencing transcription factor), SOX9 (SRY-box transcription factor 9), RhoA (Ras homolog family member A), RhoC (Ras homolog family member C), APT1 (acyl-protein thioesterase 1), SIRT1 (sirtuin 1), TLX (Tailless homolog), Phf6 (PHD finger protein 6), Ezh2 (enhancer of zeste homolog 2), PSD-9 (postsynaptic density protein 95), BCL2L2 (B-cell lymphoma 2-like 2), SNCA (alpha-synuclein), SNAP25 (synaptosomal-associated protein 25), ISCU (iron-sulfur cluster assembly enzyme), PTEN (phosphatase and tensin homolog), SPRY2 (sprouty homolog 2), MAPK (mitogen-activated protein kinase), MAP1B (microtubule-associated protein 1B), PTBP1 (polypyrimidine tract-binding protein 1), BACE1 (beta-site amyloid precursor protein cleaving enzyme 1), COX4I1 (cytochrome c oxidase subunit 4I1), and LTP (long-term potentiation).

miRNA	Localization	Major Targets	Regulated Processes	Specific Function	References
miR-132	CNS	p250GAP MeCP2	RAC1-actin signaling Activity-dependent transcription	Dendritic arborization Spine density Synaptic plasticity	[90,91]
miR-134	CNS	LIMK1	Actin filament dynamics Spine size	Spine morphology regulation Synaptic strength Impact on the LTP process	[92,93,94,95]
miR-124	CNS	REST SOX9 RhoA	Neuronal transcription Dendritic growth	Neuronal identity Differentiation Maintenance of neural morphology	[96,97,98,99,100]
miR-138	CNS	APT1 RhoC	Actomyosin contractility Palmitoylation	Spine expansion and stabilization	[101]
miR-181	CNS	SIRT1	Actin filament assembly	Dendritic branching Synaptic protein localization	[102]
miR-9	CNS	REST TLX Rho GTPases	Dendritic arborization Cytoskeletal dynamics	Dendritic development Spine formation Activity-dependent plasticity	[103]
miR-128	CNS	PHF66 EZH2	Cytoskeletal gene expression Synaptic receptor composition	Neuronal excitability Dendritic arborization	[104]
miR-125b	CNS	PSD-95	Translation of postsynaptic proteins	Spine density Synaptic stability	[105]
miR-29a/b	CNS	BCL2L2 Integrins	Cytoskeletal remodeling Neuronal survival	Dendritic maintenance Synaptic stability under stress	[106,107]
miR-153	CNS	SNCA SNAP25	Synaptic homeostasis	Prevention of protein toxicity Spine stability	[108,109]
miR-210	CNS	ISCU Mitochondrial genes	Mitochondrial function ATP production	Spine morphogenesis Activity-driven plasticity	[110]
miR-21	PNS	PTEN SPRY2	PI3K-AKT MAPK signaling Cytoskeletal remodeling	Axonal growth Regeneration Schwann cell support	[111,112]
miR-182	PNS	RAC1 Cofilin	Actin polymerization Microtubule stabilization	Axonal guidance and elongation	[113]
miR-431	PNS	KREMEN1 SPROUTY2	Wnt and MAPK signaling Growth cone remodeling	Peripheral axonal regeneration	[114]
miR-9	PNS	MAP1B Neurofilament L	Microtubule stabilization Axonal caliber	Axonal development and branching	[115]
miR-132	PNS	RASA1	GTPase signaling Neurite outgrowth	Growth cone signaling Axon elongation	[116]
miR-124	PNS	SOX9 PTBP1	Neuronal and glial differentiation	Induction of differentiation Restricts aberrant glial proliferation	[117]
miR-195	PNS	BACE1	Microtubule dynamics Local protein degradation	Growth cone motility Axonal elongation	[118]
miR-338	CNS/PNS	COX4I1 Mitochondrial genes	Mitochondrial function Local energy supply	Supports dendritic spine morphogenesis (CNS) and growth cone motility (PNS)	[119,120,121]

**Table 2:** Comprehensive summary of miRNAs investigated across different species and pain models, their targets, and the resulting effects on neuropathic pain modulation. Given the large number of abbreviations included in this table, a complete list with full definitions is provided in the abbreviations section.

miRNA	Species Tested	Pain Model	Molecular Targets	Effects on Neuropathic Pain	References
miR-1	Rat	CCI	BDNF Cx43	miR-1 was downregulated in the sciatic nerve, accompanied by upregulation of its targets BDNF and Cx43 following CCI	[122]
miR-7	Rat	SNL	VGSC β2 subunit	miR-7a overexpression in injured DRG neurons suppressed β2 subunit upregulation and normalized nociceptor hyperexcitability	[123]
miR-15	Rat	CCI	IL-1β IL-6 TNF-α AKT3	Intrathecal miR-15a agomir attenuated CCI-induced neuropathic pain and the expression of some pro-inflammatory cytokines (IL-6, IL-1β, and TNF-α), while promoting autophagy through AKT3 inhibition	[124]
	Rat	CIPN (Oxaliplatin)	BACE1	miR-15b overexpression in DRG neurons induced mechanical allodynia accompanied by downregulation of BACE1	[125]
miR-16	Rat	CCI	GRK2	Inhibition of miR-15a/16 upregulated GRK2 and suppressed p38 MAPK and NF-κB activation in CCI rats, while GRK2 silencing reversed these antinociceptive effects	[126]
miR-17 cluster	Rat	SNL	VGKC	Overexpression of some miR-17 cluster members (miR-18a/19a/19b/92a) induced mechanical allodynia, while their inhibition alleviated VGKC-dependent mechanical allodynia	[127]
miR-20	Rat	CCI	AKT3	In CCI rats, miR-20b-5p downregulation and Akt3 upregulation were observed. miR-20b-5p mimics alleviated neuropathic pain, neuroinflammation, and AKT3 expression by targeting AKT3, whereas AKT3 abrogated these effects	[128]
miR-21	Mouse	SNI	TGF-βR2 TGF-β1	Conditional deletion of miR-21 in DRG neurons promoted M2-like TGF-β-dependent antinociceptive phenotypes, and reversed mechanical allodynia	[129]
		CCI	RECK MMP9 CLDN1	miR-21 downregulated RECK, upregulated MMP9, and suppressed CLDN1 via the RECK/MMP9 pathway. Perineurial miR-21 mimics induced mechanical hypersensitivity in mice, whereas local miR-21 inhibition after nerve injury reversed it	[130]
miR-22	Rat	CCI	ENO1	circZNF609 sponges miR-22-3p, promoting pro-inflammatory factor expression and exacerbating neuropathic pain via the miR-22-3p/ENO1 axis in CCI rats	[131]
miR-23	Mouse	pSNL	CXCR4	Intrathecal miR-23a overexpression reduced spinal CXCR4 and prevented pSNL-induced neuropathic pain, whereas miR-23a knockdown induced pain-like behavior, reversible by CXCR4 inhibition	[132]
		CCI	Not studied	Metabolomics identified 283 metabolites altered in KO-CCI mice, some of which are linked to neuropathic pain	[133]
miR-26	Rat	CCI	MAPK6	MAPK6 upregulation markedly reversed the inhibitory effect of miR-26a-5p on neuropathic pain	[134]
miR-28	Rat	CCI	ZEB1	ZEB1 was upregulated in CCI rats and negatively regulated by miR-28-5p. ZEB1 overexpression promoted neuropathic pain via upregulation of COX-2, IL-6, and IL-1β	[135]
miR-29	Rat	SNI	OXTR GABA	Silencing miR-29c increased the pain threshold in SNI rats. This fact was associated with upregulation of OXTR and GABA, suggesting pain relief via the OT-GABA pathway. OXTR inhibition reversed inhibitory postsynaptic currents	[136]
miR-30	Rat	CCI	CYP24A1	miR-30b-5p alleviated neuropathic pain and neuroinflammation in CCI rats by targeting CYP24A1 and suppressing the Wnt/β-catenin pathway	[137]
miR-31	Mouse	CCI	TRAF6	miR-31-5p knockout or miPEP31 (miPEP31 peptide encoded by pri-miRNA-31) administration exacerbated pain, whereas its overexpression increased paw withdrawal threshold and latency. TRAF6, a target of miR-31-5p, mediated inflammatory responses and pain, and reducing its DRG expression alleviated pain. miR-31-5p overexpression suppressed TRAF6 and attenuated neuroinflammation	[138]
miR-32	Rat	SNL	DUSP5	Knockdown of miR-32-5p markedly reduced mechanical allodynia, heat hyperalgesia, and pro-inflammatory cytokines (IL-1β, IL-6, and TNF-α) in SNL rats. Similarly, miR-32-5p knockdown decreased cytokine production in LPS-treated spinal microglia, whereas overexpression had the opposite effect	[139]
miR-34	Rat	CCI	VAMP-2	Time-dependent downregulation of miR-34a was confirmed by qPCR after CCI, and VAMP-2 expression was significantly increased at 12 days post-CCI	[140]
	Mouse	CCI	NLRP3	miR-34c overexpression alleviated CCI-induced neuropathic pain by directly targeting the 3′-UTR of NLRP3, resulting in reduced spinal expression of NLRP3 inflammasome components and pro-inflammatory cytokines (IL-1β and IL-18)	[141]
miR-92	Rat	CCI	WNT5A	miR-92a-3p was downregulated under inflammatory and CCI conditions, contributing to enhanced Wnt5a-mediated neuroinflammation and neuropathic pain, while restoration of miR-92a-3p reduced pain behaviors	[142]
miR-93	Rat	CCI	STAT3	miR-93 overexpression attenuates neuropathic pain and reduces IL-1β, IL-6, and TNF-α in CCI rats by directly targeting STAT3. Suppression of STAT3 mediates the anti-inflammatory and analgesic effects of miR-93, while STAT3 overexpression reverses them	[143]
miR-96	Rat	CCI	Nav1.3	miR-96 mitigated neuropathic pain by downregulating Nav1.3/SCN3A. Following CCI, miR-96 levels decreased while Nav1.3/SCN3A expression increased in the DRG. Intrathecal administration of miR-96 suppressed CCI-induced Nav1.3 upregulation	[144]
miR-98	Rat	CCI	STAT3	miR-98 overexpression attenuated neuropathic pain by suppressing neuroinflammation. miR-98 reduced IL-6, IL-1β, and TNF-α levels, while STAT3 reversed these effects	[145]
miR-99	Rat	CCI	MMP13	miR-99b-3p alleviated CCI-induced mechanical allodynia and neuroinflammation by targeting MMP13, suppressing microglial pyroptosis, and enhancing autophagy in the spinal cord	[146]
miR-100	Rat	SCI	TLR4 NF-κB	miR-100 is downregulated in activated microglia and SCI, accompanied by upregulation of TLR4/NF-κB signaling, I-κB degradation, iNOS, among other. miR-100 overexpression reversed these changes, reduced microglial activation and apoptosis, and improved motor function in SCI rats	[147]
miR-101	Rat	CCI	MKP-1	miR-101 is upregulated in the SDH and microglia of CCI rats, promoting pain hypersensitivity by targeting MKP-1 and activating the MAPK pathway	[148]
	Rat	CCI	mTOR	miR-101 is downregulated in the SDH and microglia of CCI rats. miR-101 directly targets mTOR, suppressing pro-inflammatory cytokines (IL-1β, IL-6, and TNF-α), and alleviating CCI-induced mechanical and thermal hypersensitivity	[149]
miR-103	Rat	SNL	Cav1.2 α1 subunit Cav1.2 α2δ1 subunit Cav1.2 β1 subunit	miR-103 bidirectionally regulates Cav1.2 subunits, and its knockdown in naïve rats induces pain hypersensitivity	[150]
miR-106	Mouse	SNI	P2X4R	miR-106b-5p overexpression in BV2 cells reversed LPS-induced P2X4R upregulation and attenuated SNI-induced neuropathic pain by reducing P2X4R expression in the SDH. Conversely, intrathecal miR-106b-5p inhibition induced pain behaviors and increased P2X4R expression in naïve mice	[151]
miR-122	Mouse	CCI	PDK4	PDK4 expression was upregulated during neuropathic pain, and shPDK4 alleviated mechanical allodynia and thermal hyperalgesia in CCI mice. Co-administration of miR-122-5p with shPDK4 further enhanced these analgesic effects	[152]
miR-124	Rat	CCI	EZH2	miR-124-3p attenuated neuroinflammation and neuropathic pain by suppressing IL-6, IL-1β, and TNF-α expression in CCI rats. EZH2 was identified as a direct target of miR-124-3p, and its overexpression reversed the effects of miR-124-3p	[153]
	Rat	SNL	EGR1	Intrathecal miR-124-3p prevents SNL-induced neuropathic pain and upregulation of EGR1, while miR-124-3p inhibition in naïve rats induces pain and EGR1 overexpression	[154]
miR-125	Mouse	DPN	TRAF6	In db/db mice, reduced thermal and mechanical sensitivity and elevated GFAP and MCP-1 are associated with decreased miR-125a-5p. miR-125a-5p mimic increased pain sensitivity while suppressing GFAP and MCP-1 via targeting TRAF6	[155]
	Rat	CCI	SOX11	miR-125b-5p overexpression alleviated mechanical allodynia and thermal hyperalgesia in CCI rats by regulating SOX11	[156]
miR-126	Mouse	PSL	EFHD2	miR-126 was downregulated in the PSL neuropathic pain model, and its overexpression alleviated pain by suppressing inflammation via targeting EFHD2	[157]
miR-128	Rat	CCI	ZEB1	miR-128-3p attenuated CCI-induced neuropathic pain by reducing neuroinflammation. ZEB1 was identified as a direct target of miR-128-3p and was suppressed in microglia, indicating that miR-128-3p alleviates neuropathic pain via ZEB1 modulation	[158]
	Rat	SCI	AQP4	NEAT1 was significantly upregulated in SCI rats and promoted neuroinflammation by increasing IL-1β, IL-6, and TNFα levels. NEAT1 acted as a ceRNA to sponge miR-128-3p, thus relieving miR-128-3p-mediated repression of AQP4	[159]
miR-129	Rat	CCI	HMGB1	miR-129-5p was progressively downregulated in CCI rats and was associated with increased pro-inflammatory cytokine levels and pain behaviors. Overexpression of miR-129-5p alleviated neuropathic pain by suppressing HMGB1 and reducing neuroinflammation	[160]
miR-130	Rat	SCI	IGF-1	miR-130a-3p was upregulated in SCI and promoted neuropathic pain by suppressing IGF-1. Its inhibition reduced neuroinflammation and pain, indicating that miR-130a-3p aggravated SCI-induced neuropathic pain via IGF-1 repression	[161]
	Mouse	SCI	CXCL12 CXCR4	miR-130a-5p was downregulated in neuropathic pain, and its restoration alleviated CCI-induced pain by suppressing astrocyte activation and inflammation through inhibition of the CXCL12/CXCR4-RAC1-NF-κB/ERK signaling pathway	[162]
miR-132	Rat	SNI	Not studied	miR-132-3p was upregulated in the spinal cord after SNI and contributed to persistent neuropathic pain. Intrathecal inhibition of miR-132-3p reversed allodynia and pain aversion, whereas its overexpression induced pain in naïve rats, indicating a pro-nociceptive role for miR-132-3p in neuropathic pain	[163]
miR-133	Rat	DPN	VEGFR-2 p38 MAPK TRAF6 PIAS3 NF-κB MKP3	miR-133a-3p promoted diabetic neuropathic pain by activating p38 MAPK signaling in the sciatic nerve. Intraneural overexpression induced mechanical allodynia, whereas miR-133a-3p inhibition alleviated pain in diabetic rats, indicating miR-133a-3p as a potential therapeutic target for DPN	[164]
	Rat	CPSP	P2X4R	miR-133b-3p negatively correlated with allodynia in CPSP rats. Overexpression of miR-133b-3p or P2X4R knockdown in the VPL alleviated allodynia, while miR-133b-3p inhibition blocked gabapentin’s antiallodynic effect, implicating P2X4R regulation and the endogenous opioid system	[165]
miR-134	Rat	CCI	TWIST1	Overexpression of miR-134-5p was found to markedly suppress TWIST1. TWIST1 expression was progressively upregulated in CCI rats but could be inhibited in vivo. Downregulation of TWIST1 attenuated neuropathic pain progression in CCI rats through suppression of neuroinflammation	[166]
miR-135	Rat	CCI	NCKX2	SLC24A2 was downregulated in the SDH after CCI, contributing to neuropathic pain and neuroinflammation. Overexpression of SLC24A2 alleviated hyperalgesia and reduced pro-inflammatory cytokines. miR-135a-5p alleviated neuropathic pain by directly targeting and suppressing SLC24A2	[167]
	Rat	CCI	Not studied	ciRS-7 was positively correlated with neuropathic pain progression and promoted autophagy and inflammation in CCI rats. ciRS-7 acted as a sponge for miR-135a-5p, and inhibition of miR-135a-5p reduced inflammation, and pain	[168]
miR-136	Rat	CCI	IL-6R	CRNDE was upregulated in CCI rats and promoted neuropathic pain by inducing neuroinflammation, increasing IL-1, IL-6, IL-10, and TNF-α levels. CRNDE acted by sponging miR-136, which was downregulated in CCI and targeted IL6R. Loss of miR-136 enhanced IL-6R expression and neuroinflammation, mediating CRNDE-induced pain	[169]
miR-137	Rat	CCI	TNFAIP1	miR-137 was negatively regulated by XIST and its upregulation attenuated neuropathic pain in CCI rats. TNFAIP1, a target of miR-137 and a positive regulator of NF-κB-mediated inflammation, was upregulated in CCI. miR-137 inhibited TNFAIP1 expression, an effect reversed by XIST	[170]
miR-138	Rat	PHN	ROCK2	miR-138-5p was downregulated in PHN and alleviated pain by targeting ROCK2, reducing astrocyte activation and pro-inflammatory cytokines	[171]
miR-139	Mouse	SCI	MST1	miR-139-5p improved locomotor recovery, reduced pain hypersensitivity, and promoted neuronal survival in SCI mice by activating AMPKα, enhancing mitochondrial function, and inhibiting NF-κB-mediated inflammation. Its protective effects were MST1-dependent	[172]
miR-140	Rat	CCI	S1PR1	miR-140 was downregulated in CCI rats, which exhibited mechanical and thermal hyperalgesia and increased IL-1β, IL-6, and IFN-γ. Intrathecal miR-140 agomiR alleviated pain and reduced neuroinflammation by targeting S1PR1, while S1PR1 activation reversed these effects	[173]
miR-141	Rat	CCI	HMGB1	miR-141 was downregulated in CCI rats, and its overexpression alleviated neuropathic pain by suppressing HMGB1	[174]
miR-142	Mouse	SNL	HMGB1	miR-142-3p was downregulated in the DRG of SNL mice. Its overexpression alleviated neuropathic pain by directly targeting HMGB1, whose expression was inversely correlated with miR-142-3p. HMGB1 reversed these effects	[175]
	Rat	CCI	CDK5 TRPV1	miR-142-5p, downregulated in neuropathic pain, inhibited CDK5 and TRPV1 expression, reduced IL-6 and TNF levels, and improved pain thresholds. Its maturation was suppressed by m6A modification	[176]
miR-143	Rat	SNL	MOR	miR-143 downregulation mediated SNL-induced DNMT3A upregulation in DRG, leading to reduced OPRM1/MOR expression, central sensitization, and neuropathic pain. Restoration of miR-143 reversed these effects and improved morphine analgesia, whereas miR-143 inhibition in naive rats mimicked SNL-induced neuropathic pain	[177]
miR-144	Mouse	CCI	RASA1	miR-144 was downregulated in CCI-induced neuropathic pain, and its intrathecal overexpression alleviated pain and neuroinflammation by targeting RASA1. Overexpression of RASA1 reversed these protective effects	[178]
miR-145	Rat	CCI	AKT3	miR-145 alleviated neuropathic pain in CCI rats by targeting AKT3, suppressing NF-κB and mTOR signaling, and reducing inflammation and ion channel overexpression	[179]
miR-146	Mouse	SNL	TRAF6	miR-146a-5p negatively regulated TRAF6 in astrocytes, suppressing JNK activation, CCL2 expression, and neuroinflammation. Its overexpression alleviated mechanical allodynia in LPS-, TNF-α-, and SNL-induced neuropathic pain models	[180]
	Rat	CCI	IRAK1 TRAF6	miR-146a-5p was upregulated in L4-L6 DRGs and SDH after CCI. Intrathecal miR-146a-5p agomir alleviated mechanical and thermal hyperalgesia and reduced IRAK1 and TRAF6 expression, while miR-146a-5p antagomir exacerbated pain and further increased their expression	[181]
miR-148	Rat	SCIRI	SMURF2 FOXA2	miR-148a-3p promoted microglial polarization from M1 to M2 and alleviated SCIRI-induced neuropathic pain by targeting SMURF2, preventing SIRT1 ubiquitination, with FOXA2 acting as an upstream regulator	[182]
miR-150	Rat	CCI	TLR5	miR-150 overexpression alleviated neuropathic pain and reduced COX-2, IL-6, and TNF-α levels in CCI rats. miR-150 suppressed TLR5 expression, whereas TLR5 overexpression reversed the analgesic effects of miR-150	[183]
miR-151	Rat	CCI	NOTCH2	miR-151a-3p was downregulated in CCI rats and alleviated neuropathic pain by repressing NOTCH2. circ_0005075 counteracted miR-151a-3p, restoring NOTCH2 expression and reversing its protective effects	[184]
miR-154	Rat	CCI	TLR5	miR-154-5p was negatively regulated by XIST. XIST overexpression promoted neuropathic pain in CCI rats, whereas miR-154-5p upregulation reversed this effect. TLR5 was identified as a target of miR-154-5p, which suppressed its expression, and miR-154-5p mitigated TLR5-induced pain	[185]
miR-155	Rat	bCCI	SGK3	CCAT1 expression was decreased in the SDH, DRG, hippocampus, and ACC of bCCI rats, which showed cold allodynia after 14 days. CCAT1 suppressed miR-155 and upregulated SGK3 in NGF-differentiated PC12 cells. In bCCI rats, miR-155 was increased, whereas SGK3 was downregulated	[186]
	Rat	DPN	NRF2	miR-155 regulated MNCV, SNCV, angiogenesis, and inflammation in DPN rats via NRF2. Silencing miR-155 or restoring NRF2 promoted cell proliferation, inhibited apoptosis, and suppressed inflammation in vitro. In vivo, miR-155 inhibition improved nerve conduction, enhanced angiogenesis, and reduced inflammation, whereas NRF2 restoration reversed these effects	[187]
miR-181	Rat	SNL	TNFAIP1	miR-181b was identified as a direct target of TNFAIP1. Upregulation of miR-181b attenuated the pro-apoptotic and pro-inflammatory effects of lncRNA p21 and TNFAIP1, likely via the AKT/CREB pathway. In vivo, lncRNA p21 knockdown in SNL rats alleviated neuropathic pain, as indicated by increased PWMT and PWTL	[188]
	Rat	CCI	Not studied	miR-181c-5p expression was progressively downregulated in CCI rats. ExomiR-181c-5p was efficiently delivered to microglia, where it inhibited pro-inflammatory cytokine secretion. Intrathecal administration of ExomiR-181c-5p alleviated neuropathic pain and neuroinflammation in CCI rats	[189]
miR-182	Rat	CCI	PI3K/AKT	Intrathecal administration of LV-mediated miR-182 alleviated neuropathic pain in CCI rats, as indicated by increased PWMT and PWTL, reduced pro-inflammatory cytokines (TNF-α, IL-1β, and IL-6), and reduced M1 microglial polarization. These neuroprotective effects were completely abolished by 740Y-P (PI3K/AKT activator)	[190]
miR-183	Rat	CCI	mTOR VEGF receptors	miR-183 directly targeted mTOR and regulated VEGF expression through mTOR. Downregulation of miR-183 or activation of the mTOR/VEGF pathway upregulated AMPA receptors, indicating that miR-183 modulates neuropathic pain via the mTOR/VEGF/AMPA signaling axis	[191]
	Rat	CCI	MAP3K4	miR-183 was downregulated in CCI rats. Overexpression of miR-183 alleviated mechanical and thermal hyperalgesia and reduced pro-inflammatory cytokines (IL-6, IL-1β, and COX-2). MAP3K4 was identified as a direct target of miR-183, and its suppression mediated the inhibitory effects on pro-inflammatory cytokine expression	[192]
miR-184	Mouse	DPN	CCL1	miR-184-5p was downregulated in the SDH after STZ-induced DPN. Intrathecal miR-184-5p agomir alleviated neuropathic pain, while miR-184-5p antagomir induced pain behaviors in naive mice. CCL1 was confirmed as a direct target of miR-184-5p, and CCR8 expression was also increased after STZ. miR-184-5p overexpression suppressed CCL1/CCR8 levels, whereas its inhibition increased them	[193]
miR-185	Rat	CCI	MyD88 CXCR4 NLRP3	miR-185-5p upregulation alleviated hyperalgesia, inhibited glial activation, and reduced IL-1β, IL-6, and TNF-α in CCI rat spinal cord. miR-185-5p suppressed MyD88, CXCR4, and NLRP3 inflammasome in microglia, while knockdown of MyD88/CXCR4 enhanced its anti-inflammatory effects. Moreover, miR-185-5p reduced microglia-induced neuronal apoptosis and promoted neuronal survival	[194]
miR-187	Mouse	IR	P2X7R	Ischemia–reperfusion decreased PWT and PWL via downregulation of miR-187-3p and upregulation of P2X7R. Mimic-187 restored miR-187-3p, suppressed P2X7R/IL-1β/caspase-1, and preserved PWT/PWL, while inhibitor-187 or P2X7R activation reversed these effects	[195]
miR-190	Mouse	DPN	VGLUT2	In DPN spinal tissues, miR-190a-5p was downregulated and VGLUT2 upregulated. miR-190a-5p overexpression or VGLUT2 inhibition alleviated pain behaviors and reduced IL-1β and IL-6 levels	[196]
miR-193	Mouse	DPN	HMGB1	In STZ-induced diabetic mice, miR-193a was downregulated and HMGB1 upregulated in the lumbar spinal dorsal horn. miR-193a suppressed HMGB1 expression, alleviated neuropathic pain, and reduced peripheral neuroinflammation	[197]
miR-194	Rat	CCI	FOXA1	miR-194 was downregulated in CCI rats, and its overexpression alleviated neuroinflammation and neuropathic pain. FOXA1 was identified as a direct target of miR-194, and its restoration reversed these effects. miR-194 reduced COX-2, IL-6, and IL-10 levels	[198]
miR-195	Rat	SNL	ATG14	miR-195 was upregulated in spinal microglia after SNL, promoting LPS-induced IL-1β, TNF-α, and iNOS expression. miR-195 overexpression increased mechanical and cold hypersensitivity, while its inhibition reduced pain and enhanced microglial autophagy, suppressing neuroinflammation. Autophagy blockade reversed the protective effects of miR-195 inhibition	[199]
	Rat	CCI-IoN	PACHTED1	miR-195 was upregulated and PACTHED1 downregulated in a CCI-IoN rat model. Luciferase assays confirmed miR-195 targets PATCHED1. miR-195 overexpression exacerbated facial pain, which was reversed by PATCHED1 upregulation	[200]
miR-199	Rat	NLBP	TRPV1	miR-199 negatively correlated with TRPV1 and directly targeted its 3′UTR. In vitro and rat NLBP models, miR-199 modulation alleviated or exacerbated neuronal injury	[201]
miR-200	Rat	CCI	BRD3	miR-200a-3p was downregulated in CCI rats in a time-dependent manner. Its overexpression alleviated mechanical and thermal hyperalgesia by targeting BRD3. BRD3 downregulation inhibited neuropathic pain, while BRD3 overexpression reversed the effects of miR-200a-3p on NF-κB signaling and pain	[202]
miR-202	Rat	bCCI	RAP1	miR-202 was downregulated in the SDH, but unchanged in the DRG, hippocampus, and anterior cingulate cortex of bCCI rats. RAP1A was identified as a direct target of miR-202 and was upregulated in the SDH. miR-202 overexpression increased hindpaw pain thresholds, an effect reversed by RAP1A overexpression	[203]
miR-203	Rat	bCCI	RAP1	miR-203 was markedly downregulated in the SDH, but not in the DRG, hippocampus, or anterior cingulate cortex of bCCI rats. RAP1A protein was upregulated in the SDH, and miR-203 targeted its 3′-UTR, reducing RAP1A expression in neuron-like cells	[204]
miR-204	Rat	CCI	BRD4	miR-204-5p was downregulated in CCI rats, accompanied by reduced mechanical and thermal pain thresholds and increased pro-inflammatory factors. miR-204-5p overexpression increased PWT and PWL and decreased pro-inflammatory markers	[205]
miR-206	Rat	CCI	BDNF	miR-206 was downregulated in the DRG after CCI, while BDNF was upregulated. miR-206 mimics reduced pain, inflammation (TNF-α, IL-1β, and IL-6), BDNF expression, and MEK/ERK activation. BDNF overexpression reversed these effects, whereas ERK inhibition (by U0126) mimicked the miR-206 action	[206]
miR-212	Rat	CCI	Nav1.3	In CCI rats, PWT and PWL were reduced and IL-1β, IL-6, and TNF-α were increased, while miR-212-3p was downregulated. Intrathecal miR-212-3p agomir restored PWT/PWL, reduced inflammatory cytokines, decreased BAX and cleaved caspase-3, increased BCL-2, and directly targeted the 3′-UTR of NaV1.3, whose expression was downregulated by miR-212-3p	[207]
	Rat	SCI	PTEN	In SCI rats, miR-212-3p was downregulated, with increased PTEN and cleaved caspase-3 and decreased p-AKT, p-mTOR, and Bcl-2. AgomiR-212-3p reversed these effects by targeting PTEN, suppressing apoptosis, and restoring cell viability and signaling in LPS-injured PC12 cells	[208]
miR-214	Rat	SNL	CSF1	SNL reduced miR-214-3p via DNMT3A-mediated promoter hypermethylation. miR-214-3p targeted CSF1, and its overexpression reduced CSF1, IL-6, astrocyte activation, and pain, while DNMT inhibition restored miR-214-3p and alleviated these effects	[209]
miR-216	Rat	CCI	KDM3A	miR-216a-5p overexpression inhibited IL-1β, IL-6, and TNF-α, suppressed microglial infiltration, and inactivated the Wnt/β-catenin pathway, reducing neuroinflammation. KDM3A was identified as a downstream target, and its knockdown alleviated neuropathic pain, while KDM3A overexpression reversed miR-216a-5p effects	[210]
miR-218	Rat	CCI	STAT3	Downregulation of miR-218 in CCI rats alleviated mechanical allodynia, thermal hyperalgesia, and pro-inflammatory cytokine release. miR-218 directly targeted SOCS3, regulating its mRNA and protein levels. miR-218 inhibition inactivated STAT3 signaling, blocking pro-inflammatory gene expression	[211]
miR-221	Rat	CCI	SOCS1	miR-221 was overexpressed in the SDH of CCI rats. Intrathecal miR-221 inhibition reduced mechanical allodynia, thermal hyperalgesia, and TNF-α, IL-1β, and IL-6 levels. SOCS1 was identified as a direct target, and miR-221 inhibition suppressed SOCS1, NF-κB, and p38 MAPK activation, whereas SOCS1 knockdown reversed these effects	[212]
miR-221 miR-222	Rat	SCI	LASS2	Upregulation of the miR-221/222 cluster was associated with injury-induced Schwann cell phenotypic modulation, promoting proliferation and migration. miR-221/222 directly targeted LASS2 by binding its 3′-UTR, reducing LASS2 mRNA and protein levels. LASS2 silencing mimicked miR-221/222 overexpression, while LASS2 knockdown reversed the inhibitory effects of miR-221/222 suppression	[213]
miR-223	Mouse	CCI	NLRP3	miR-223 overexpression alleviated CCI-induced neuropathic pain by reducing apoptosis and inflammatory factor expression. miR-223 directly targeted NLRP3, suppressing NLRP3 inflammasome components (ASC, caspase-1, IL-1β, and IL-18) and promoting macrophage polarization toward the anti-inflammatory M2 phenotype	[214]
	Mouse	CCI-IoN	MKNK2	miR-223-3p was downregulated and MKNK2 upregulated in TGs of CCI-ION mice. miR-223-3p overexpression alleviated trigeminal neuropathic pain by targeting MKNK2, reducing proinflammatory cytokines, and inhibiting MAPK/ERK signaling	[215]
miR-299	Mouse	SCI	PHLPP1	miR-299a-5p was markedly downregulated in the spinal cord after SCI. miR-299a-5p agomir attenuated, whereas its antagomir exacerbated, inflammation, oxidative stress, and injury by directly targeting PHLPP1 and activating AMPK signaling. PHLPP1 overexpression or AMPK inhibition abolished the protective effects of miR-299a-5p	[216]
miR-320	Rat	CCI	FTX	FTX was downregulated in CCI rats and directly targeted miR-320a. FTX overexpression alleviated neuropathic pain by reducing PWT, PWL, and neuroinflammation, effects reversed by miR-320a upregulation. RUNX2, a downstream target of miR-320a, was suppressed in CCI rats and restored by FTX, but this restoration was attenuated by miR-320a	[217]
miR-323	Rat	CCI-IoN	Kv2.1	miR-323-3p was upregulated in injured trigeminal ganglia, driving neuropathic pain by suppressing Kv2.1 and increasing neuronal excitability. Its expression was promoted through PRMT2-mediated H3R8 methylation and FOXA2 activation. Inhibition of miR-323-3p restored Kv2.1 and reduced mechanical hypersensitivity	[218]
miR-330	Rat	CCI	PDC4	DGCR5 was downregulated in CCI rats, while miR-330-3p was upregulated. DGCR5 overexpression alleviated mechanical and thermal hyperalgesia and suppressed IL-1β, IL-6, and TNF-α, by sponging miR-330-3p. PDC4, a downstream target of miR-330-3p, was regulated by DGCR5 through this axis, contributing to reduced neuroinflammation and neuropathic pain	[219]
miR-331	Rat	SCI	RAP1A	miR-331-3p was downregulated in the spinal cord of SCI rats. miR-331-3p overexpression improved locomotor function, reduced tissue damage, neuronal apoptosis, and inflammation. RAP1A was identified as a downstream target, and its upregulation reversed the protective effects of miR-331-3p by promoting apoptosis and inflammation, while miR-331-3p inhibited RAP1A signaling	[220]
miR-339	Rat	CPSP	MOR	In the CPSP rats, OPRM1/MOR was downregulated and miR-339-5p upregulated. miR-339-5p directly targeted the 3′-UTR of OPRM1/MOR, suppressing MOR mRNA expression	[221]
miR-340	Rat	CCI	RAP1A	miR-340-5p was downregulated in spinal cord of CCI rats. Its overexpression restored PWT and PWL, reduced inflammation, and decreased COX-2, IL-1β, TNF-α, and IL-6 levels. RAP1A was identified as a direct target, and RAP1A overexpression reversed the protective effects of miR-340-5p	[222]
miR-362	Mouse	CCI	BAMBI	LncRNA Miat knockdown alleviated mechanical and cold hyperalgesia and reduced neuroinflammation in CCI mice. Mechanistically, lncRNA Miat sponged miR-362-3p to upregulate BAMBI, promoting neuropathic pain development	[223]
miR-378	Rat	CCI	EZH2	miR-378 was downregulated in CCI rats, and its overexpression alleviated mechanical and thermal hyperalgesia. EZH2, a downstream target negatively regulated by miR-378, reversed these protective effects when upregulated	[224]
miR-381	Rat	CCI	HMGB1 CXCR4	miR-381 was downregulated in CCI rats, and its overexpression alleviated mechanical and thermal hyperalgesia and reduced IL-6, IL-10, and TNF-α. HMGB1 and CXCR4, predicted and confirmed as direct targets of miR-381, were upregulated in CCI rats, and their silencing suppressed neuropathic pain progression	[225]
miR-382	Mouse	SCI	NRG1	After SCI, lncRNA Ftx and NRG1 were downregulated while miR-382-5p was upregulated, promoting microglial inflammation. Ftx acted as a ceRNA, sponging miR-382-5p to relieve its inhibition of NRG1	[226]
	Rat	CCI	DUSP1	miR-382-5p was upregulated, and DUSP1 downregulated in CCI rats. Inhibiting miR-382-5p alleviated mechanical and thermal hypersensitivity and suppressed pro-inflammatory responses by restoring DUSP1 expression, whereas DUSP1 knockdown abolished these protective effects	[227]
miR-384	Rat	CCI	Nav1.3	miR-384-5p was downregulated in CCI rats. Its overexpression alleviated mechanical allodynia and heat hyperalgesia, suppressed spinal microglial inflammation, and reduced pro-inflammatory cytokines. Nav1.3 was identified as a direct target of miR-384-5p	[228]
miR-388	Rat	CCI	TRPV1	TRPV1 and NECAB2 were upregulated in the SDH of CCI rats and cooperatively promoted mechanical allodynia, thermal hyperalgesia, and microglial inflammation via mGluR5, p-ERK1/2, and Ca^2+^ signaling. TRPV1 was identified as a direct target of miR-338-3p, and its overexpression reversed the protective effects of miR-338-3p on neuropathic pain	[229]
miR-411	Rat	SCI	IL-18	miR-411 suppressed IL-18 expression and inhibited the JNK pathway, thereby reducing microglial inflammation in vitro. In SCI rats, miR-411 attenuated inflammation and apoptosis, preserved axonal integrity, and enhanced locomotor recovery, as reflected by increased BBB scores	[230]
miR-423	Rat	SNL	SNHG4	SNHG4 knockdown alleviated neuropathic pain, reducing mechanical and thermal hyperalgesia and suppressing neuroinflammation by decreasing IL-6, IL-12, and TNF-α while increasing IL-10. Bioinformatics identified miR-423-5p as a target of SNHG4, which negatively regulated miR-423-5p. miR-423-5p was downregulated in SNL rats, and its loss contributed to neuropathic pain, an effect reversed by SNHG4 silencing	[231]
miR-200 miR-429	Rat	CCI	ZEB1	miR-200b and miR-429 were downregulated in spinal cords and microglia of CCI rats. Their overexpression alleviated neuropathic pain by increasing PWT and PWL and suppressed neuroinflammation through downregulating IL-6, IL-1β, and TNF-α. ZEB1 was identified as a direct target of both miRNAs, upregulated in CCI rats, and knockdown of ZEB1 reduced pain, whereas inhibition of miR-200b/miR-429 reversed this effect	[232]
miR-448	Rat	CCI	SIRT1	miR-448 was upregulated in CCI rats, and its inhibition alleviated mechanical allodynia, thermal hyperalgesia, and increased IL-1β, IL-6, and TNF-α levels. SIRT1 was identified as a direct target of miR-448, mediating its pro-nociceptive effects through neuroinflammation	[233]
miR-488	Rat	CCI	ROCK1	miR-488-3p was downregulated and ROCK1 upregulated in the DRGs of CCI rats. miR-488-3p overexpression alleviated mechanical and thermal hypersensitivity, suppressed pro-inflammatory cytokines, and increased anti-inflammatory factors. ROCK1 was identified as a direct target of miR-488-3p, and its upregulation partially reversed these effects	[234]
miR-489	Rat	SNL	TET1	miR-489-3p was downregulated in SDH neurons after SNL, leading to upregulation of DEK, which recruited TET1 to the BDNF, GRM5, and STAT3 promoters and enhanced their transcription. Inhibition of miR-489-3p in naïve rats induced similar effects, while miR-489-3p overexpression in SNL rats attenuated allodynia and reversed these transcriptional changes	[235]
miR-494	Rat	SCI	RAD23B	lncRNA SNHG12 was upregulated in the DRGs of SNI rats and localized mainly in the cytoplasm of PC12 cells. SNHG12 knockdown attenuated neuropathic pain, increasing PWT and PWL and reducing IL-1β, IL-6, and TNF-α levels. Mechanistically, SNHG12 regulated RAD23B expression by sponging miR-494-3p, and its expression was controlled by KLF2	[236]
miR-495	Rat	DPN	USP15	miR-497 is downregulated and USP15 is upregulated in DRG neurons of STZ-induced DNP rats. miR-497 alleviates DPN by suppressing USP15, which promotes NRF2 ubiquitination and dysregulates G6PD expression	[237]
miR-500	Rat	CIPN (Paclitaxel) SNL	GAD67	CIPN or SNL-induced neuropathic pain impaired GABAergic synaptic function in SDH neurons by reducing GAD67 expression. This effect was mediated by upregulation of miR-500, which directly targets the GAD1 gene. Inhibition or knockout of miR-500 restored GABAergic synapses and alleviated pain hypersensitivity	[238]
miR-503	Mouse	DPN	SEPT9	miR-503-5p expression was reduced in the spinal cord of DPN mice and in high glucose-treated astrocytes. Restoration of miR-503-5p alleviated neuropathic pain behaviors and suppressed astrocytic activation and inflammation by directly targeting SEPT9	[239]
miR-506	Rat	tBPI	CCL2	miR-506-3p was downregulated in the spinal cord after tBPI, accompanied by activation of the CCL2/CCR2/NF-κB inflammatory pathway. Overexpression of miR-506-3p reduced neuronal apoptosis and microglial inflammation by directly targeting CCL2 and suppressing CCR2/NF-κB signaling	[240]
miR-539	Rat	CCI	NR2B	CCI altered miRNA expression in the ACC, with miR-539 markedly downregulated in the contralateral ACC and concomitant upregulation of NR2B. Restoration of miR-539 or pharmacological inhibition of NR2B in the contralateral ACC significantly attenuated mechanical allodynia	[241]
miR-181a miR-203 miR-541*	Rat	bCCI	Not studied	bCCI induced miRNA dysregulation along the pain pathway. miR-341 was selectively upregulated in the DRG, whereas miR-203, miR-181a-1*, and miR-541* were downregulated in the SDH	[242]
miR-543	Rat	SCI	NF-κB	SCI reduced miR-543-5p expression in the rat spinal cord. Restoration of miR-543-5p suppressed NF-κB signaling and inflammatory mediator expression while enhancing nerve regeneration-related factors, leading to improved functional recovery after injury	[243]
miR-544	Rat	CCI	STAT3	miR-544 alleviated neuropathic pain CCI rats by suppressing several pro-inflammatory cytokines through direct targeting of STAT3. This protective effect was reversed by lncRNA XIST, which promoted neuropathic pain progression by sponging miR-544 and upregulating STAT3	[244]
miR-547	Rat	CCI	IL-33	CCI reduced miR-547-5p expression and enhanced IL-33/ST2 signaling in the DRG and SDH, contributing to neuropathic pain. Restoration of miR-547-5p suppressed IL-33/ST2 activation and alleviated pain hypersensitivity, whereas miR-547-5p inhibition mimicked CCI-induced changes	[245]
miR-590	Mouse	DPN	RAP1A	miR-590-3p was downregulated in DPN mice. Restoration of miR-590-3p alleviated pain behaviors, reduced proinflammatory cytokines, and inhibited immune cell infiltration. miR-590-3p targeted RAP1A, and RAP1A overexpression partially reversed the inhibitory effects of miR-590-3p on T cell proliferation and migration	[246]
miR-665	Rat	CCI	SOCS1	In CCI rats, miR-665 was upregulated in the spinal cord, contributing to decreased paw withdrawal threshold and latency and increased pro-inflammatory cytokines. miR-665 directly targeted SOCS1, suppressing its expression. Downregulation of miR-665 alleviated pain and inflammation, effects that were reversed by SOCS1 knockdown	[247]
miR-672	Rat	CIPN (Bortezomib)	REEP6	miR-672-5p was downregulated in dorsal horn neurons of rats with bortezomib-induced neuropathic pain. Restoration of miR-672-5p reduced neuronal hyperexcitability and mechanical allodynia, while miR-672-5p knockdown induced pain and increased excitatory synaptic activity in naïve rats. Mechanistically, miR-672-5p directly targeted REEP6, and REEP6 upregulation contributed to neuronal hyperexcitability and pain	[248]
miR-709	Mouse	SCI	NKAP	Microglial pyroptosis peaked on day 7 after SCI and was exacerbated by Treg cell depletion, leading to impaired motor recovery. Conversely, Treg cell infiltration promoted functional recovery by inhibiting microglial pyroptosis. Mechanistically, Treg cell-derived exosomes delivered miR-709, which targeted NKAP to suppress microglial pyroptosis and inflammation. Exosomes enriched with miR-709 further reduced inflammation and enhanced motor recovery	[249]
miR-719	Mouse	CCI	Nav1.1	lncRNA Snhg16 was upregulated in CCI mice and promoted neuropathic pain by sponging miR-719, leading to increased SCN1A/Nav1.1 expression. Inhibition of miR-719 or upregulation of SCN1A/Nav1.1 reversed the effects of Snhg16 on pain behaviors and neuroinflammation	[250]
miR-1906	Rat	CCI	NF-κB TLR4	Administration of miR-1906 agomir alleviated mechanical and thermal hypersensitivity in CCI-induced neuropathic pain and reduced levels of pro-inflammatory mediators. Mechanistically, miR-1906 suppressed spinal AKT/mTOR/PI3K signaling and downregulated TLR-4 and NF-κB expression	[251]

## Data Availability

No new data were created or analyzed in this study. Data sharing is not applicable to this article.

## Abbreviations

The following abbreviations are used in this manuscript:

3′ UTR	3′ untranslated region
740 Y-P	740YPDGFR
ACC	Anterior cingulate cortex
AGO	Argonaute
AGO2	Argonaute 2
AKT	Protein kinase B
AKT3	RAC-alpha serine/threonine-protein kinase 3
AMPA	α-amino-3-hydroxy-5-methyl-4-isoxazolepropionic acid
AMPKα	AMP-activated protein kinase alpha
APT1	Acyl-protein thioesterase 1
AQP4	Aquaporin 4
ATG14	Autophagy related 14
ATP	Adenosine triphosphate
ASC	Apoptosis-associated speck-like protein containing a CARD
BACE1	β-site amyloid precursor protein cleaving enzyme 1
BAMBI	BMP and activin membrane-bound inhibitor
BAX	Bcl-2-like protein 4
BBB	Basso, Beattie and Bresnahan locomotor rating scale
bCCI	Bilateral chronic constriction injury
BCL2	B-cell lymphoma 2
BCL2L2	B-cell lymphoma 2-like 2
BDNF	Brain-derived neurotrophic factor
BRD3	Bromodomain containing 3
BRD4	Bromodomain containing 4
Ca^2+^	Calcium ion
Cav1.2	Voltage-gated calcium channel subunit 1.2
ceRNA	Competing endogenous RNA
CCI	Chronic constriction injury
CCI-IoN	Chronic constriction injury of infraorbital nerve
CCL1	Chemokine (C-C motif) ligand 1
CCL2	Chemokine (C-C motif) ligand 2
CCR2	C-C chemokine receptor type 2
CCR4-NOT	Carbon catabolite repression 4-negative on TATA-less complex
CCR8	C-C chemokine receptor type 8
CDK5	Cyclin-dependent kinase 5
CIPN	Chemotherapy-induced peripheral neuropathy
ciRS	Circular RNA
CSF1	Colony stimulating factor 1
CLDN1	Claudin 1
CNS	Central nervous system
COX-2	Cyclooxygenase 2
COX4I1	Cytochrome c oxidase subunit 4 isoform 1
CPSP	Chronic post-surgical pain
CREB	cAMP response element-binding protein
CRPS	Complex regional pain syndrome
Cx43	Connexin 43
CXCL12	C-X-C motif chemokine ligand 12
CXCR4	C-X-C chemokine receptor type 4
CX3CL1	C-X3-C motif chemokine ligand 1
CYP24A1	Cytochrome P450 family 24 subfamily A member 1
DGCR5	DiGeorge syndrome critical region 5
DGCR8	DiGeorge syndrome critical region 8
Dicer	RNase III endonuclease
DNA	Deoxyribonucleic acid
DNMT3A	DNA methyltransferase 3 alpha
DPN	Diabetic peripheral neuropathy
DRG	Dorsal root ganglion
DUSP1	Dual specificity phosphatase 1
DUSP5	Dual specificity phosphatase 5
EFHD2	EF-hand domain family member D2
EGR1	Early growth response 1
ENO1	Enolase 1
ERK	Extracellular signal-regulated kinase
EZH2	Enhancer of zeste homolog 2
FOXA2	Forkhead box A2
GABA	Gamma-aminobutyric acid
GAD1	Glutamate decarboxylase 1
GAD67	Glutamate decarboxylase 67 kDa isoform
G6PD	Glucose-6-phosphate dehydrogenase
GRK2	G protein-coupled receptor kinase 2
GRM5	Glutamate metabotropic receptor 5
GTPase	Guanosine triphosphatase
GW182	Glycine-tryptophan protein of 182 kDa
HDL	High-density lipoprotein
HMGB1	High mobility group box 1
hnRNP	Heterogeneous nuclear ribonucleoprotein
I-κB	Inhibitor of κB
IASP	International Association for the Study of Pain
IGF-1	Insulin-like growth factor 1
IL-1β	Interleukin 1 beta
IL-10	Interleukin 10
IL-12	Interleukin 12
IL-18	Interleukin 18
IL-33	Interleukin 33
IL-6	Interleukin 6
IL-6R	Interleukin 6 receptor
iNOS	Inducible nitric oxide synthase
IR	Ischemia–reperfusion
IRAK1	Interleukin-1 receptor-associated kinase 1
ISCU	Iron-sulfur cluster assembly enzyme
JNK	c-Jun N-terminal kinase
KLF2	Krüppel-like factor 2
KREMEN1	Kringle containing transmembrane protein 1
Kv2.1	Voltage-gated potassium channel subunit 2.1
LASS2	Longevity assurance homolog 2
LIMK1	LIM domain kinase 1
lncRNA	Long non-coding RNA
LPS	Lipopolysaccharide
LTP	Long-term potentiation
MAP1B	Microtubule-associated protein 1B
MAP3K4	Mitogen-activated protein kinase kinase kinase 4
MAPK	Mitogen-activated protein kinase
MAPK6	Mitogen-activated protein kinase 6
MeCP2	Methyl CpG-binding protein 2
MEK	Mitogen-activated protein kinase kinase
miRNA	MicroRNA
miRNA*	Passenger strand of microRNA duplex
MKNK2	MAP kinase-interacting serine/threonine-protein kinase 2
MKP-1	MAP kinase phosphatase 1
MKP3	MAP kinase phosphatase 3
MMP13	Matrix metallopeptidase 13
MMP9	Matrix metallopeptidase 9
MNCV	Motor nerve conduction velocity
MOR/OPRM1	Mu opioid receptor
mRNA	Messenger RNA
MST1	Mammalian sterile 20-like kinase 1
mTOR	Mechanistic target of rapamycin
MyD88	Myeloid differentiation primary response 88
Nav1.1/SCN1A	Voltage-gated sodium channel alpha subunit 1.1
Nav1.3/SCN3A	Voltage-gated sodium channel alpha subunit 1.3
NCKX2/SLC24A2	Sodium/calcium/potassium exchanger
ncRNA	Non-coding RNA
NEAT1	Nuclear enriched abundant transcript 1
NF-κB	Nuclear factor kappa-light-chain-enhancer of activated B cells
NGF	Nerve growth factor
NGS	Next-generation sequencing
NKAP	NF-κB activating protein
NLBP	Neck and low back pain
NLRP3	NOD-, LRR- and pyrin domain-containing protein 3
NMDA	N-Methyl-D-aspartate
NRF2	Nuclear factor erythroid 2-related factor 2
NRG1	Neuregulin 1
OT	Oxytocin
OXTR	Oxytocin receptor
p-ERK1/2	Phospho-ERK1/2
PACHTED1	PATCHED domain-containing protein 1
p250GAP	GTPase-activating protein p250
P2X4R	P2X4 receptor
P2X7R	P2X7 receptor
p38 MAPK	p38 mitogen-activated protein kinase
p68/p72	RNA helicases p68 and p72
PACT	Protein activator of the interferon-induced protein kinase
PDC4	Pyruvate decarboxylase 4
PDCD4	Programmed cell death protein 4
PDK4	Pyruvate dehydrogenase kinase 4
PHF6	Plant homeodomain finger protein 6
PHLPP1	PH domain and leucine-rich repeat protein phosphatase 1
PHN	Post-herpetic neuralgia
PIAS3	Protein inhibitor of activated STAT 3
PI3K	Phosphoinositide 3-kinase
PIAS3	Protein inhibitor of activated STAT3
PNS	Peripheral nervous system
pre-miRNA	Precursor microRNA
pri-miRNA	Primary microRNA transcripts
PSD-95	Postsynaptic density protein 95
PSL	Partial sciatic ligation
pSNL	Partial spinal nerve ligation
PTBP1	Polypyrimidine tract-binding protein 1
PTEN	Phosphatase and tensin homolog
PWMT	Paw withdrawal mechanical threshold
PWTL	Paw withdrawal thermal latency
qRT-PCR	Quantitative real-time polymerase chain reaction
RAC1	Ras-related C3 botulinum toxin substrate 1
RAD23B	RAD23 homolog B, nucleotide excision repair protein
Ran-GTP	Ras-related nuclear protein bound to GTP
RAP1	Ras-related protein 1
RAP1A	Ras-related protein 1A
RASA1	RAS p21 protein activator 1
RECK	Reversion-inducing cysteine-rich protein with kazal motifs
REEP6	Receptor accessory protein 6
REST	RE1-silencing transcription factor
RhoA	Ras homolog family member A
RhoC	Ras homolog family member C
RISC	RNA-induced silencing complex
RNA	Ribonucleic acid
RNS	Reactive nitrogen species
ROCK1	Rho-associated protein kinase 1
ROCK2	Rho-associated protein kinase 2
ROS	Reactive oxygen species
RUNX2	Runt-related transcription factor 2
S1PR1	Sphingosine-1-phosphate receptor 1
SCI	Spinal cord injury
SCIRI	Spinal cord ischemia–reperfusion
SGK3	Serum/glucocorticoid regulated kinase 3
SIRT1	Sirtuin 1
Smad	Mothers against decapentaplegic homolog
SMURF2	SMAD ubiquitination regulatory factor 2
SNAP25	Synaptosome associated protein 25
SNCA	α-synuclein
SNHG12	Small nucleolar RNA host gene 12
SNHG16	Small nucleolar RNA host gene 16
SNHG4	Small nucleolar RNA host gene 4
SNCV	Sensory nerve conduction velocity
SNI	Spared nerve injury
SNL	Spinal nerve ligation
SOCS1	Suppressor of cytokine signaling 1
SOCS3	Suppressor of cytokine signaling 3
SOX9	SRY-box transcription factor 9
SOX11	SRY-box transcription factor 11
SPRY2	Sprouty homolog 2
STAT3	Signal transducer and activator of transcription 3
tBPI	Total brachial plexus injury
TET1	Ten-eleven translocation methylcytosine dioxygenase 1
TGF-β	Transforming growth factor beta
TGF-β1	Transforming growth factor beta 1
TGF-βR2	Transforming growth factor beta receptor 2
TLR	Toll-like receptor
TLR4	Toll-like receptor 4
TLR5	Toll-like receptor 5
TLX	Tailless homolog
TN	Trigeminal neuralgia
TNFAIP1	Tumor necrosis factor alpha-induced protein 1
TNF-α	Tumor necrosis factor alpha
TRAF6	TNF receptor associated factor 6
TRBP	TAR RNA-binding protein
TRP	Transient receptor potential
TRPV1	Transient receptor potential vanilloid 1
Treg	Regulatory T cell
TWIST1	Twist family BHLH transcription factor 1
U0126	1,4-diamino-2,3-dicyano-1,4-bis(2-aminophenylthio)butadiene
USP15	Ubiquitin carboxyl-terminal hydrolase 15
VAMP-2	Vesicle-associated membrane protein 2
VEGF	Vascular endothelial growth factor
VEGFR-2	Vascular endothelial growth factor receptor 2
VGCC	Voltage-gated calcium channel
VGKC	Voltage-gated potassium channel
VGLUT2	Vesicular glutamate transporter 2
VGSC	Voltage-gated sodium channel
VPL	Ventral posterolateral nucleus
WNT5A	Wnt family member 5A
XIST	X-inactive specific transcript
XPO5	Exportin 5
ZEB1	Zinc finger E-Box binding homeobox 1
